# Social vulnerability and cancer risk from air toxins in Louisiana: a spatial analysis of environmental health disparities

**DOI:** 10.3389/fpubh.2025.1601868

**Published:** 2025-05-20

**Authors:** Sadie Smith, Sahithya Sakhamuri, Chloe M. Guidry, Gabriela Mustata Wilson

**Affiliations:** Louisiana Center for Health Innovation, Informatics Research Institute, University of Louisiana at Lafayette, Lafayette, LA, United States

**Keywords:** air toxins, cancer, Louisiana, Cancer Alley, social vulnerability

## Abstract

**Introduction:**

Louisiana faces significant environmental health challenges due to elevated air toxicity near industrial sites. The state hosts over 300 manufacturing facilities, more than 150 petrochemical plants, and 15 refineries, which, although economically beneficial, pose significant health risks to surrounding communities. Exposure to industrial emissions has been linked to respiratory conditions such as asthma, reproductive disorders, kidney damage, and various cancers. An 85-mile stretch along the Mississippi River, commonly referred to as “Cancer Alley,” has long been associated with elevated cancer rates, particularly among communities with high social vulnerability.

**Methods:**

This study examines the relationship between social vulnerability, cancer incidence, and cancer risk using publicly available datasets, including air toxics data from the United States Environmental Protection Agency (EPA) and cancer incidence rates from the Louisiana Tumor Registry (LTR). Linear regression, interaction analyses, and geographically weighted regression were applied to assess how environmental and socioeconomic factors jointly influence cancer risk.

**Results:**

Results reveal that cancer incidence was associated with elevated air toxins and compounded by social factors such as minority status, low income, and single-parent households. Notably, some regions exhibited a counterintuitive negative association between air toxins and cancer incidence, which may be due to data limitations, including the use of older air quality data, latency in cancer development, or underreporting.

**Discussion:**

These findings underscore the importance of strengthening environmental regulations, real-time air quality monitoring, and community-based public health initiatives to reduce cancer disparities and support affected communities in Louisiana.

## 1 Introduction

Environmental hazards present serious and persistent public health challenges in Louisiana, disproportionately affecting marginalized communities and exacerbating longstanding health and social inequities (1, 2). The state is home to a high concentration of petrochemical plants, chemical manufacturing facilities, and refineries. Near these industrial sites are many communities that are known as fence-line communities (3). While fence-line communities can be found worldwide, those along the Gulf Coast of the United States face prolonged exposure to air, noise, and water pollution generated by facilities such as petrochemical plants (3). These communities are often predominantly low-income or racial/ethnic minority populations who bear a disproportionate burden of environmental hazards compared to non-marginalized groups (4, 5). The intersection of these factors has led to disparities in cancer incidence and mortality rates, particularly in areas such as Cancer Alley, a region spanning 11 parishes between Baton Rouge and New Orleans. While most Cancer Alley residents are white, those living in the fence-line communities directly bordering the processing plants are predominantly black (6).

Research has consistently demonstrated that exposure to environmental pollutants increases the risk of various cancers, including lung, liver, and colorectal cancers (7, 8). For example, recent studies on air pollutant exposure, specifically ambient fine particulate matter (PM_2.5_), have demonstrated that fence-line communities in Louisiana experience systematic and disproportionate exposure to these harmful pollutants, a trend that mirrors similar issues in other communities throughout the United States (9–11). People of color are also more likely to be disproportionately affected by consumer-generated pollution (12, 13). Hazardous industrial sites located near these communities’ further compound environmental injustice (14, 15). The correlation between increased exposure to hazardous pollutants and higher cancer rates is especially evident in locations with elevated levels of air toxicity, which coincide with regions that have the most vulnerable populations, especially those with higher social vulnerability (16).

From a public health perspective, policy reforms should incorporate both emissions data and population health outcomes. A continued lack of policy enforcement allows industrial emissions to increase (17)—this is particularly critical in Louisiana, where toxic air emissions are prevalent along its borders and throughout the state. As new refineries are developed, there is a noticeable absence of public health initiatives to monitor the well-being of residents and insufficient protection for those living near environmental hazards (18).

Addressing these gaps requires a comprehensive approach that considers both environmental exposures and the underlying social determinants of health. Communities with higher levels of poverty, lower educational attainment, and inadequate healthcare infrastructure often face greater challenges in mitigating environmental exposures, leading to disproportionate health outcomes (8, 19), such as elevated cancer risks (20). A more integrated understanding of these factors is essential for designing effective public health interventions and environmental policies.

This study aims to address this gap by examining the intersection of cancer incidence, cancer risk due to air toxin exposure, and social vulnerability across Louisiana’s nine public health regions. Specifically, this study seeks to answer the following research questions:

1. How does cancer incidence and cancer risk from air toxin exposure correlate across Louisiana’s nine public health regions?
2. To what extent do social vulnerability factors, such as income level, education level, and minority status, exacerbate environmental health risks?
3. What regional disparities exist, and how do they relate to industrial site locations?

To address these questions, a multifaceted analytical approach was employed, integrating statistical modeling, spatial analysis, and interaction effects to assess the relationships between cancer risk from air toxins, social vulnerability, and cancer rates. By providing a detailed assessment of cancer incidence patterns and their association with environmental and social determinants, this study seeks to inform future public health policies and interventions to reduce cancer disparities in Louisiana.

## 2 Methods

### 2.1 Study design and public health regions

This study examined cancer incidence, cancer risk from air toxin exposure, and social vulnerability factors across Louisiana’s nine public health regions, which are administrative units defined by the Louisiana Department of Health for delivering public health services and leadership. A cross-sectional environmental study design was employed to investigate how these variables intersect to influence cancer rates at the regional level.

### 2.2 Data sources and variables

Multiple publicly available datasets were integrated for this analysis, covering cancer incidence, environmental exposures, and social vulnerability indicators. Table 1 summarizes these sources and key variables.

**Table 1:** Data sources used in the analysis.

Variable	Data source	Description	Reference
Cancer incidence	Louisiana Tumor Registry (LTR), 2011–2020	LTR’s most recent 10-year cancer incidence data report includes cancer diagnoses from 2011 through 2020. All cancers’ combined incidence rates were used and are reported per 100,000 population.	(40) https://sph.lsuhsc.edu/louisiana-tumor-registry/data-usestatistics/monographs-publications/cancer-incidence-in-louisiana-by-census-tract-2024/
Cancer risk from air toxins	Environmental Protection Agency (EPA) National Air Toxics Assessment (NATA) 2011	The NATA analyzes air toxin concentrations and hazardous air pollutants across the USA and estimates the associated health risks from these pollutants. Cancer risk is quantified as the total risk per million individuals.	(41) https://www.epa.gov/national-air-toxics-assessment/2011-nata-assessment-results
Social vulnerability	2018 Centers for Disease Control (CDC) Agency for Toxic Substance and Disease Registry’s (ATSDR) Social Vulnerability Index (SVI)	Social vulnerability refers to the demographic and socioeconomic factors that adversely affect communities that encounter hazards and other community-level stressors.	(42) https://www.atsdr.cdc.gov/place-health/php/svi/index.html
Industrial sites (Power Plants)	World Resources Institute (WRI) Global Power Plant Database (GPPD)	The GPPD is a comprehensive and publicly accessible resource that provides detailed information about power plants worldwide. The database includes information about the size, location, fuel type, and operational status of power plants.	(43) https://www.wri.org/research/global-database-power-plants

The age variables (aged 65 and older and aged 17 and younger) were removed due to cancer incidence being age-adjusted. Additionally, variables within the “Housing Type and Transportation” theme and the limited English variable in the SVI were not included due to the high number of zeros in the data. For simplicity, cancer risk due to air toxins is referred to as air toxins, per capita income is referred to as income, no high school diploma is referred to as education and racial and ethnic minorities are referred to as minorities. For all analyses, the following independent variables were used: air toxins, poverty, unemployed, income, education, disabled, single parent, and minority. Cancer incidence is the outcome variable.

### 2.3 Statistical analyses

SPSS Statistics v. 26 (21) was used for regression and interaction analyses. Before conducting the linear regression analysis, exploratory data analysis (EDA) was performed to gain a deeper understanding of the data’s distributions, relationships, and potential issues. The key steps in the EDA for all variables included examining skewness, kurtosis, box plots, and histograms.

To ensure the assumptions of linear regression were met and to improve model fit, log 10 transformation was applied to air toxins. After the transformation, the normality of residuals and homoscedasticity were rechecked using diagnostic plots. The linear regression assumptions were then verified to ensure they were properly met. Given the nature of the variables, multicollinearity was assessed between all the variables, and none demonstrated extreme correlation. In the regression analysis, the first model included each variable independently, while the second model included the interaction terms (air toxins * SVI variable).

### 2.4 Geospatial analysis

Spatial analysis and map generation were performed in ArcGIS Pro 3.4 (22). Cancer incidence, air toxins, and the locations of power plants were mapped at the census tract level and overlaid with regional boundaries to visualize geographic patterns. A heat map was created to illustrate the distribution of power plant megawatt capacity. A spatial autocorrelation analysis was conducted for air toxins, and geographically weighted regression (GWR) was applied to assess spatially varying relationships.

Given the spatial nature of air pollution, the bisquare function for the local weighting scheme was selected to reflect local environmental conditions that may not adhere to census tract boundaries. Biquare weighting gives more weight to nearby locations and less weight to those farther away, which inherently considers spatial decay and aligns with pollution spread. Air toxins were log 10 transformed in the GWR as well. Before conducting the GWR, an exploratory regression analysis was performed to determine the best model for each parish and the entire state of Louisiana. Due to multicollinearity, the GWR could not be performed for certain regions. Therefore, the exploratory regression helped to identify the best variables to include in the model. Poverty was excluded from the GWR analysis due to multicollinearity. However, the income variable was included, which effectively captures economic vulnerability. For census tracts with missing SVI data, the values were substituted with 0.0001 to ensure compatibility with ArcGIS, as the software requires all entries to be numerical. There were 8 missing values in the SVI dataset. Although this approach maintained computational compatibility, it may introduce minor bias in spatial modeling. Future sensitivity analyses are warranted to evaluate the impact of such imputation on model robustness.

### 2.5 Data exclusion criteria

Louisiana contains 1,148 census tracts, of which 935 were included in the LTR dataset, which did not include any census tracts from Tensas parish (*n* = 3). Due to the absence of cancer incidence data, Tensas parish was not included in the analysis. Twenty-seven tracts were excluded from the LTR dataset because they contained military bases. Since military personnel are likely to have different exposure histories compared to the general population, these tracts were not included in the analysis. Consequently, a total of 908 census tracts from the LTR were included in the analysis.

When comparing the LTR with the SVI and NATA datasets, census tracts were excluded due to missing cancer incidence data (*n* = 193) or military designation (*n* = 27). These exclusions were distributed across the nine regions as follows:

- Region 1: 131 eliminated tracts and 4 military tracts.
- Region 2: 9 eliminated tracts and 1 military tract.
- Region 3: 6 eliminated tracts.
- Region 4: 4 eliminated tracts.
- Region 5: 5 eliminated tracts.
- Region 6: 4 eliminated tracts and 10 military tracts.
- Region 7: 18 eliminated tracts and 10 military tracts.
- Region 8: 15 eliminated tracts.
- Region 9: 1 eliminated tract and 2 military tracts.

The SVI 2018 dataset included 1,140 census tracts, and the 2011 NATA dataset contained 1,192 tracts. After aligning these datasets with the tracts in the LTR, 232 census tracts were excluded from the SVI dataset (*n* = 12 removed due to having values of 999, indicating invalid data) and 284 from the NATA dataset (*n* = 64 removed due to invalid census tract numbers of 0000). These exclusions occurred because these tracts did not match the census tracts included in the LTR.

## 3 Results

### 3.1 Geographic distribution of cancer incidence, air toxins, and industrial sites

**Figure 1:**
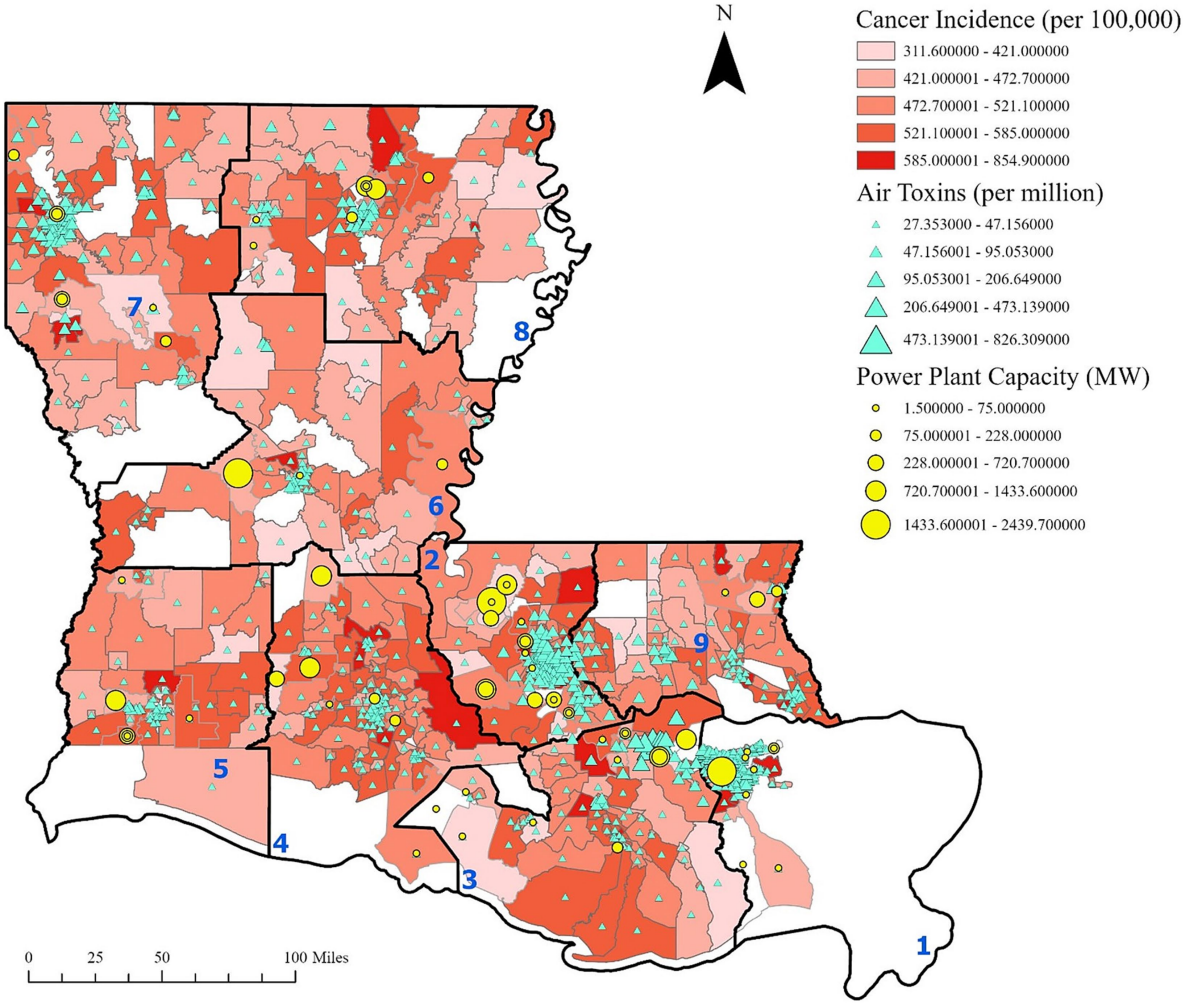
Cancer incidence, cancer risk from air toxins and power plant capacity. Cancer incidence is based on the 10-year estimate from 2011 to 2020 provided by the LTR. Power plant capacity is reported in megawatts. Regions are labeled in blue. This figure illustrates the presence of industrial sites and clustering of cancer risk from air toxins within Cancer Alley and other regions, with elevated cancer rates observed in the surrounding areas.

Geospatial analysis revealed a notable geographic clustering of cancer incidence and air toxins throughout Louisiana. Figure 1 illustrates concentrations of air toxins across the state, with particularly elevated levels in Cancer Alley and surrounding regions. Notably, cancer risk from air toxins reached a high of 826.31 cases per million, over 27 times the EPA’s acceptable threshold of 30 cases per million. The classification of Region 1 as a high-risk area is based on available data; however, it is important to note that certain census tracts in Region 1 have missing cancer incidence data, which are represented by the white areas in Figure 1. These areas reflect either unavailable or incomplete cancer data, rather than an actual absence of cancer cases. Therefore, the missing data in certain areas may affect the completeness of the overall assessment.

**Figure 2:**
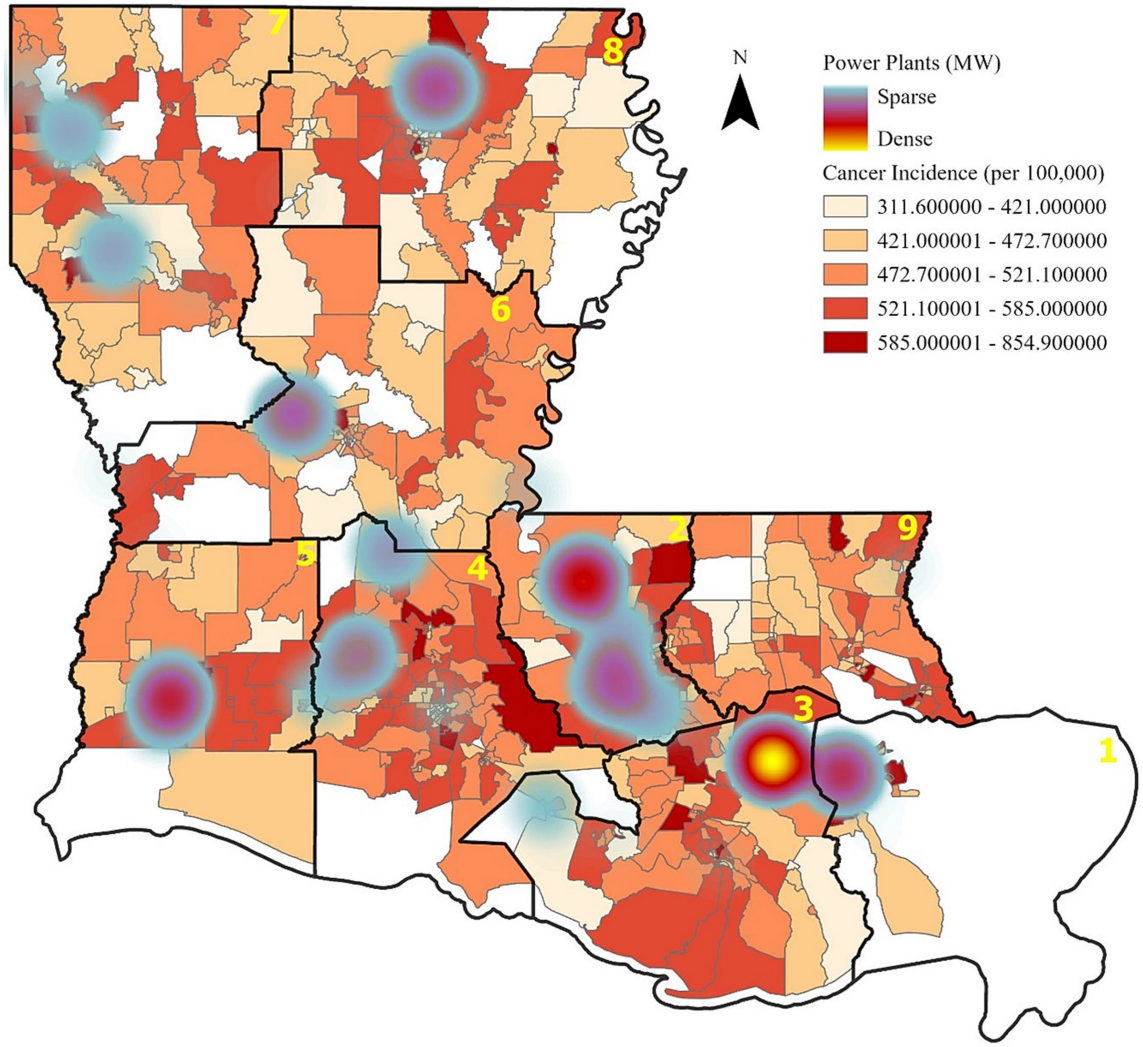
Cancer incidence and power plant capacity. Increased cancer rates, as indicated by the darker red areas, also are apparent near the more densely located power plants.

**Figure 3:**
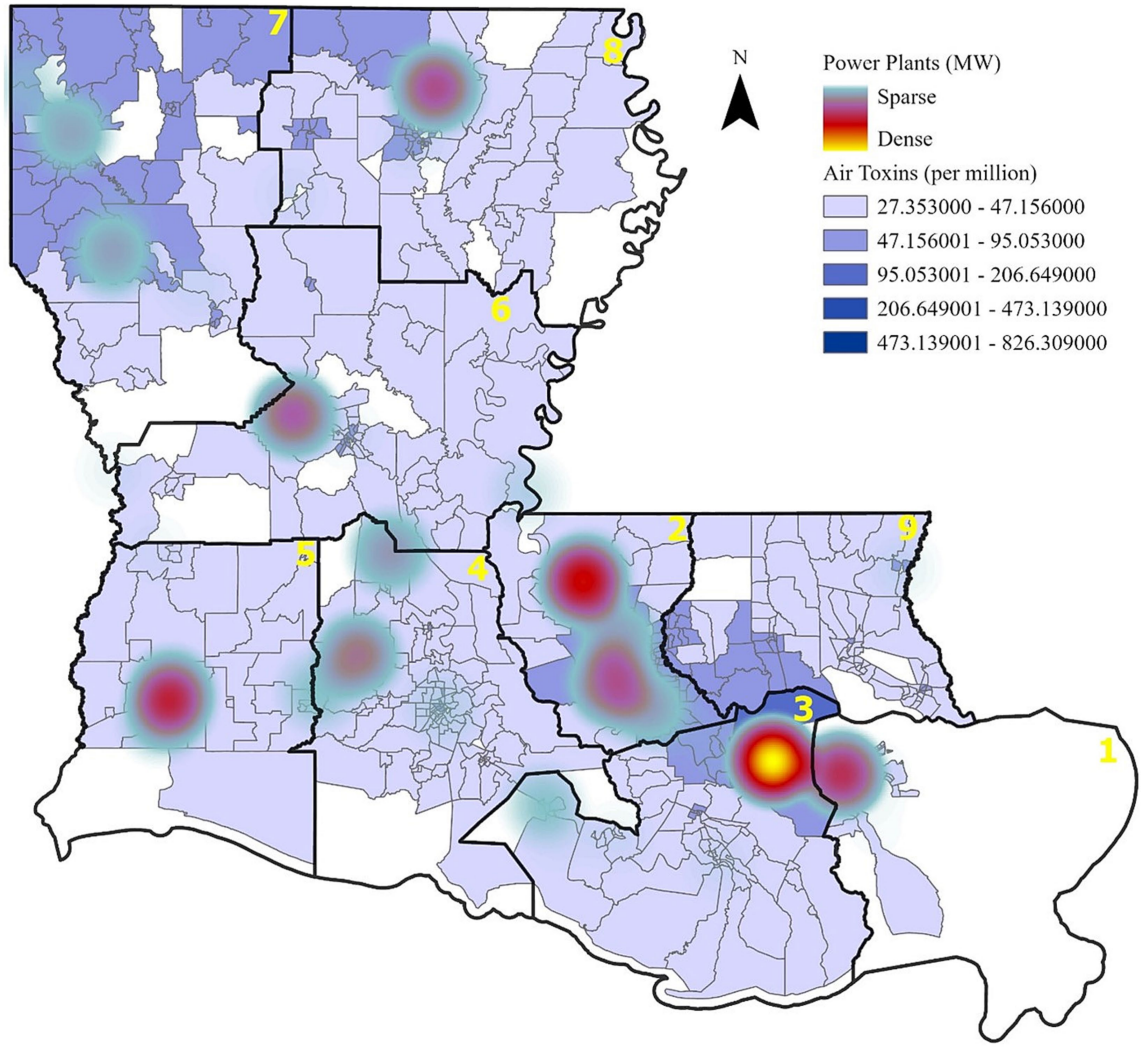
Cancer risk from air toxins and power plant capacity. Areas with higher industrial emissions correspond to elevated cancer risks due to air pollution.

Further analysis shows that power plant generation capacity is densely concentrated in Regions 1, 2, and 3, aligning with areas of higher cancer incidence and cancer risk from air toxins (Figures 2, 3). These patterns suggest a potential link between industrial emissions and increased cancer rates in adjacent communities.

### 3.2 Spatial autocorrelation of air toxins

Spatial analysis confirmed a strong and statistically significant clustering of air toxins across Louisiana. Moran’s Index was 0.597 (*z*-score: 34.43, *p* < 0.00005), indicating that air toxin concentrations are not randomly distributed but spatially correlated, with high-exposure areas clustered near industrial sites. This finding highlights regional patterns of concentrated environmental risk and supports the need for spatially targeted interventions.

### 3.3 Geographically weighted regression (GWR) findings

**Figure 4:**
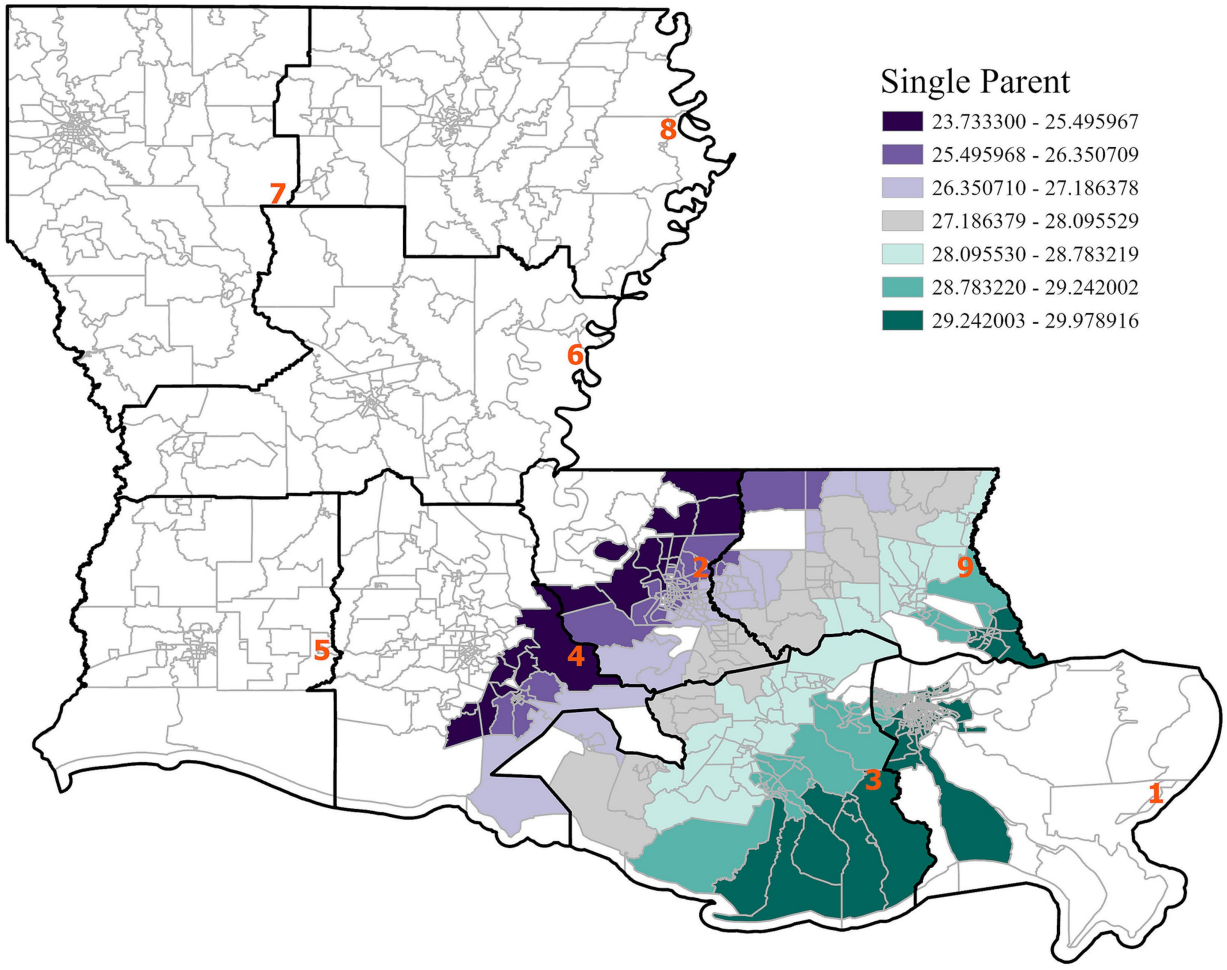
GWR analysis for the state of Louisiana, illustrating the spatial variation in the regression coefficient for single parent households. A positive association between single parent households and cancer incidence is observed.

The geographically weighted regression (GWR) analysis examined spatial relationships between cancer incidence, air toxins, and social vulnerability variables across multiple regions of Louisiana. Table 2 summarizes the significant relationships identified in the GWR analysis, while Figure 4 through 13 display region-specific maps illustrating the spatial distribution of regression coefficients. The analysis revealed considerable variability in the associations between cancer incidence and the independent variables, with some regions showing negative coefficients. This variability may reflect differences in pollution exposure levels within regions, as areas located farther from industrial sites tend to show lower exposure, which could result in inverse associations. Moreover, cancer incidence may be influenced by other unmeasured social determinants of health, such as healthcare access, housing quality, and occupational risks, all of which could mediate or confound the relationships observed.

**Table 2:** Statistically significant variables in the GWR and the direction of the association.

Location	Significant variables (+/−)	Coefficient range	Adjusted *R*^2^
Overall Louisiana	Single Parent (+)	23.73–29.98	0.02
	Air Toxins (−)		
Region 1	Air Toxins (+)	783.69–911.02	0.18
	Income (+)	194.13–232.73	
	Education (+)	112.16–128.78	
	Minority (+)	170.30–228.27	
Region 2	None		
Region 3	None		
Region 4	Minority (+)	80.06–114.23	0.13
	Air Toxins (−)		
Region 5	None		
Region 6	None		
Region 7	Disability (+)	60.86–99.05	0.07
	Education (−)		
Region 8	Income (+)	201.66–223.16	0.12
	Education (+)	148.35	
	Minority (−)		
Region 9	Single Parent (+)	44.86–94.40	0.15
	Air Toxins (−)		

Across the state, single-parent households were positively associated with cancer incidence, particularly in southeastern Louisiana. This finding suggests that regions with higher concentrations of single-parent households may experience elevated cancer risks due to socioeconomic barriers such as limited access to healthcare, lower income, and increased physical and mental stress. Statewide, air toxins were negatively associated with cancer incidence, although this result may be driven by regional differences and unmeasured confounders, as indicated by a low adjusted *R*^2^ of 0.02. Since the cancer incidence data covers 2011–2020 and the air toxin exposure data is from 2011, the full latency effect of carcinogenic exposure may not yet be fully captured. This finding may also reflect residual confounding from unmeasured variables such as smoking or healthcare access, and potential underreporting in high-risk regions. As supported by prior studies (e.g., (23)), delayed cancer manifestation following environmental exposure remains a known challenge in exposure-disease modeling.

**Figure 5:**
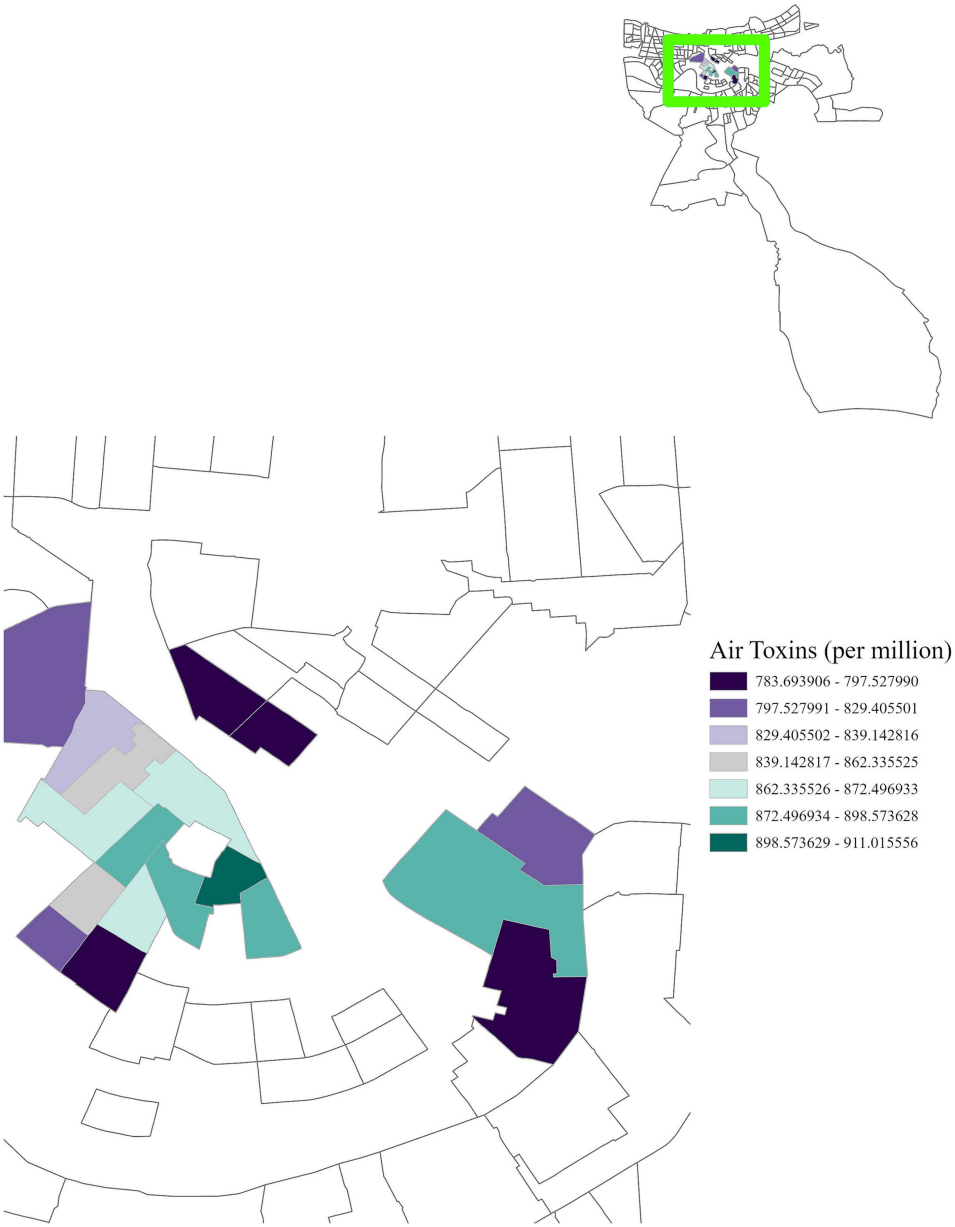
GWR analysis for Region 1, illustrating the spatial variation in the regression coefficient for cancer risk due to air toxins. In the highlighted area, for each one-unit increase in cancer risk per million, cancer incidence increases by 783.69 to 911.02 cases per 100,000 population.

**Figure 6:**
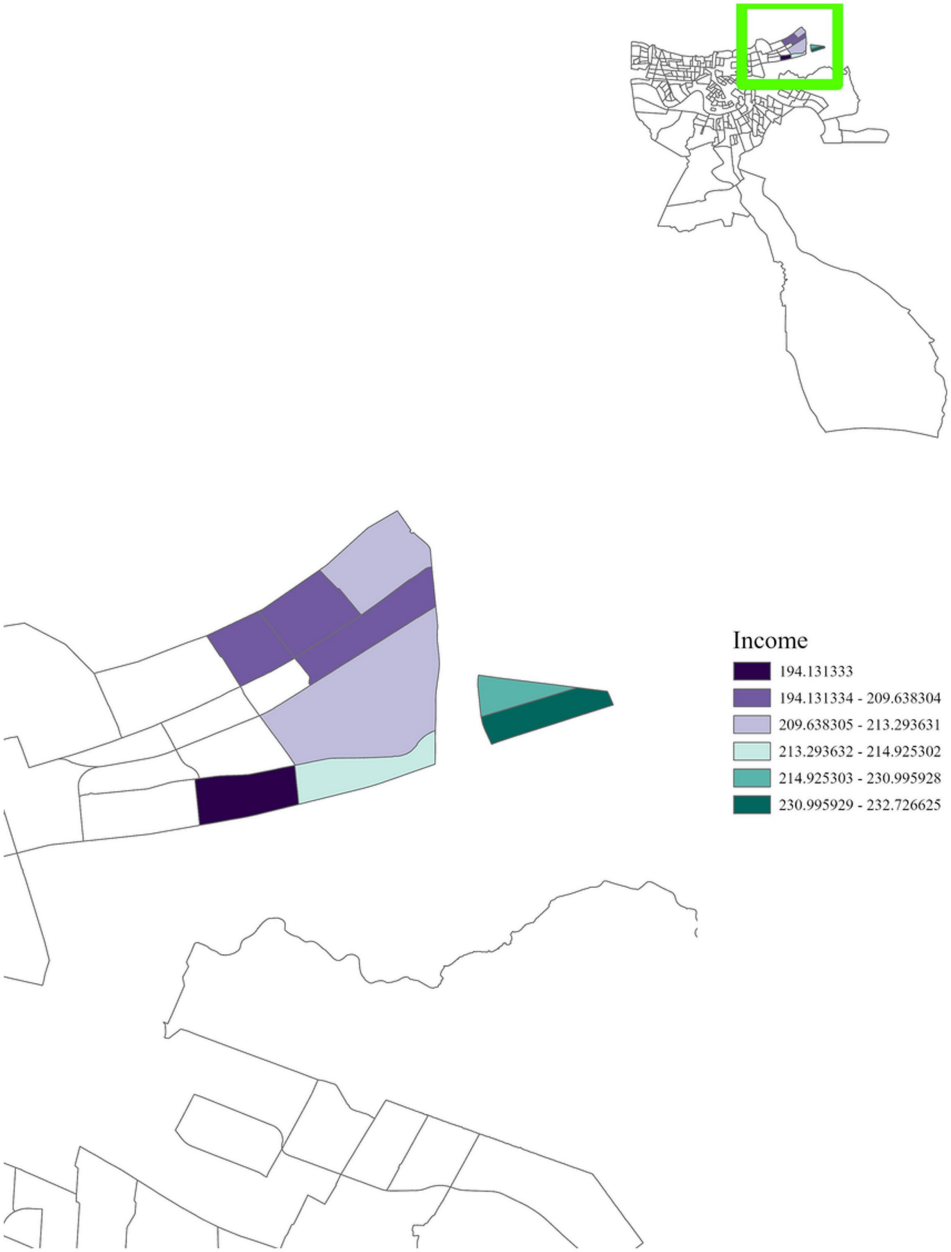
GWR analysis for Region 1, illustrating the spatial variation in the regression coefficient for income. The highlighted area reveals a positive correlation with cancer incidence, with areas close to densely concentrated industrial sites exhibiting a significant relationship.

**Figure 7:**
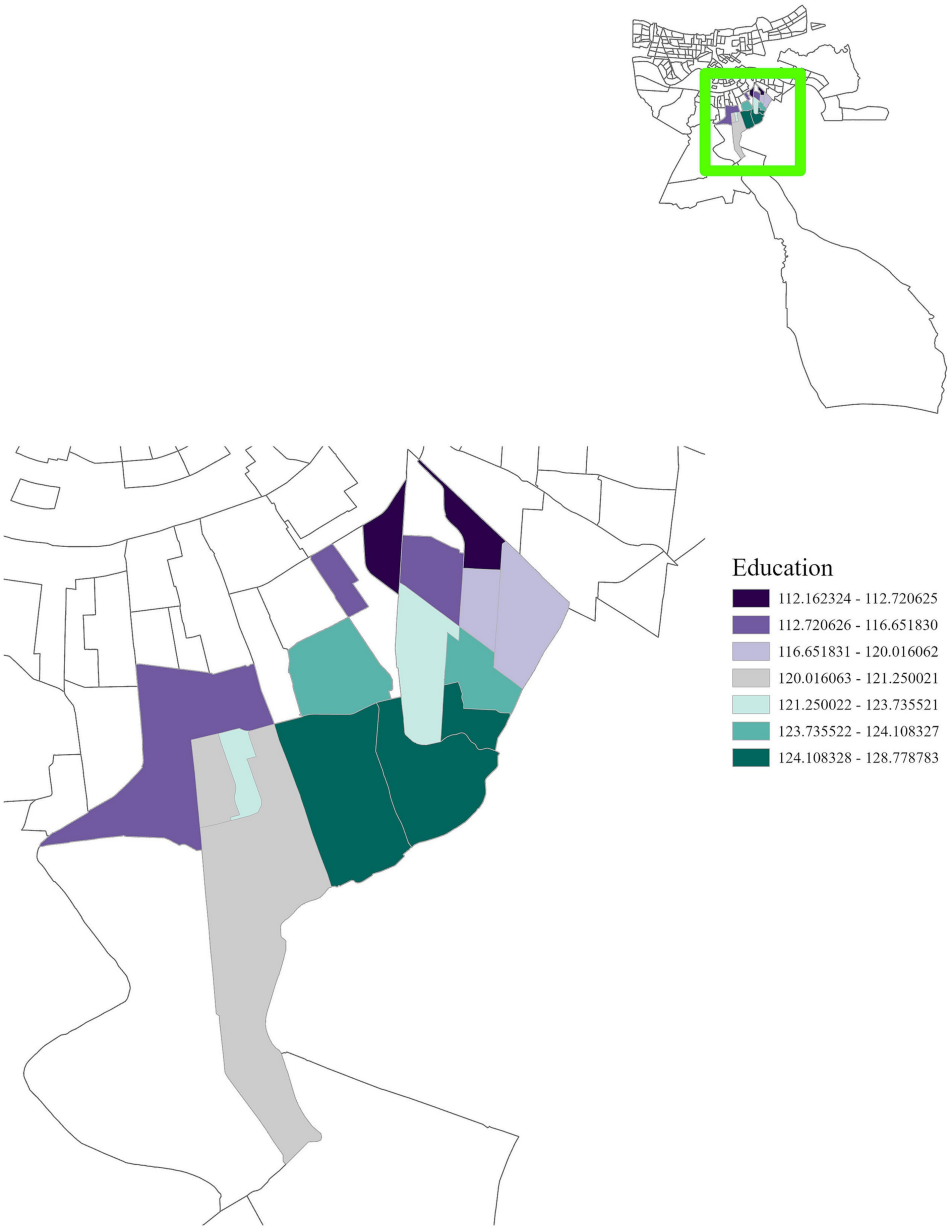
GWR analysis for Region 1, illustrating the spatial variation in the regression coefficient for education. This analysis shows a positive correlation between cancer incidence and education, emphasizing the importance of interventions to improve educational attainment in this region.

**Figure 8:**
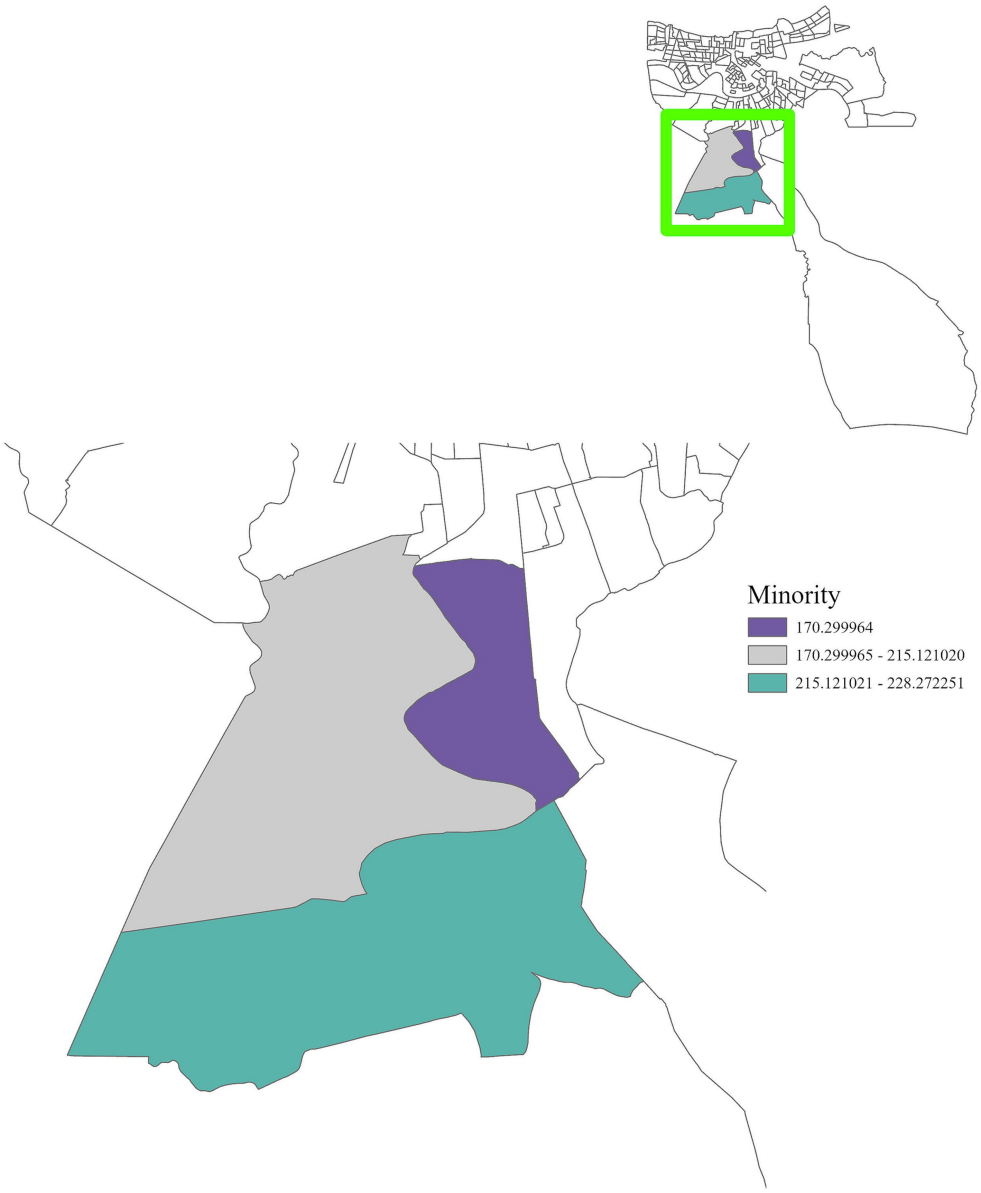
GWR analysis for Region 1, illustrating the spatial variation in the regression coefficient for minority populations. The highlighted area shows a positive correlation with cancer incidence, underscoring the importance of targeted interventions for racial and minority groups.

**Figure 9:**
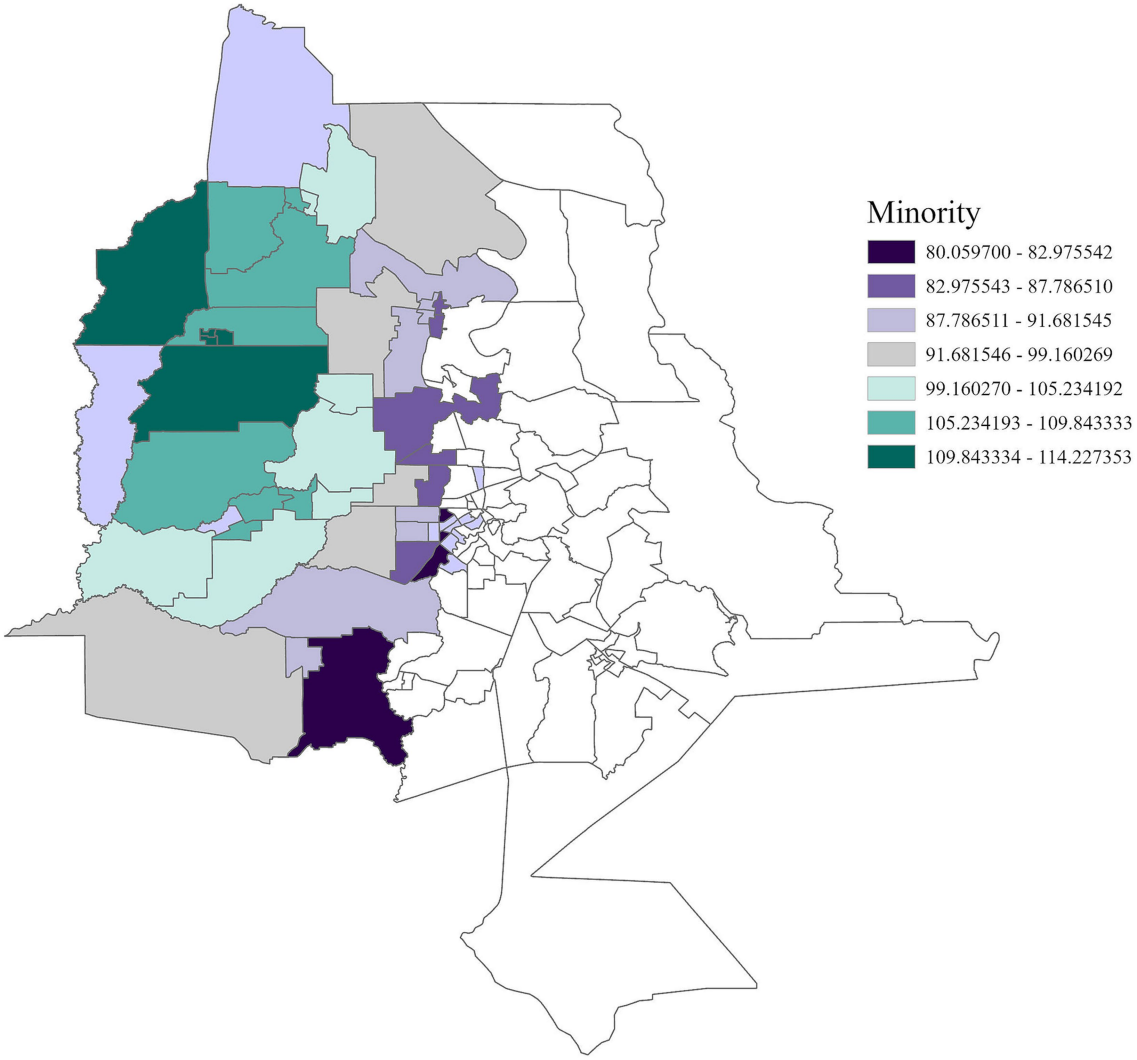
GWR analysis for Region 4, illustrating the spatial variation in the regression coefficient for racial and ethnic minority status. As vulnerability associated with minority status increases by one unit, cancer incidence rises by 80.06 to 114.23 cases per 100,000 across this region.

Regionally, distinct patterns were observed. In Region 1, cancer incidence was positively associated with air toxins, income, race, and education (Figures 5–8), with an adjusted *R*^2^ of 0.18, suggesting that in areas near industrial facilities, higher socioeconomic vulnerability may amplify the adverse health effects of environmental exposures. In addition, Region 4 exhibited a significant positive association between minority status and cancer incidence (Figure 9), highlighting racial and ethnic disparities in cancer outcomes and underscoring the need for targeted cancer prevention and treatment services within these communities (adjusted *R*^2^ = 0.13). Region 4 also showed a negative relationship between air toxins and cancer incidence, possibly related to latent effects of exposure, underreporting of cases, or other structural health determinants. No significant associations were found for Regions 2, 3, 5, and 6. In Regions 2 and 3, a negative adjusted *R*^2^ value was observed, which suggests a potential model misfit. This warrants further investigation, including an analysis accounting for additional confounding variables to better understand the relationship between socioeconomic characteristics, air pollution, and cancer incidence. For Regions 5 and 6, the models may have been affected by limited data or minimal exposure variability, indicating a need or more comprehensive data collection in these regions.

**Figure 10:**
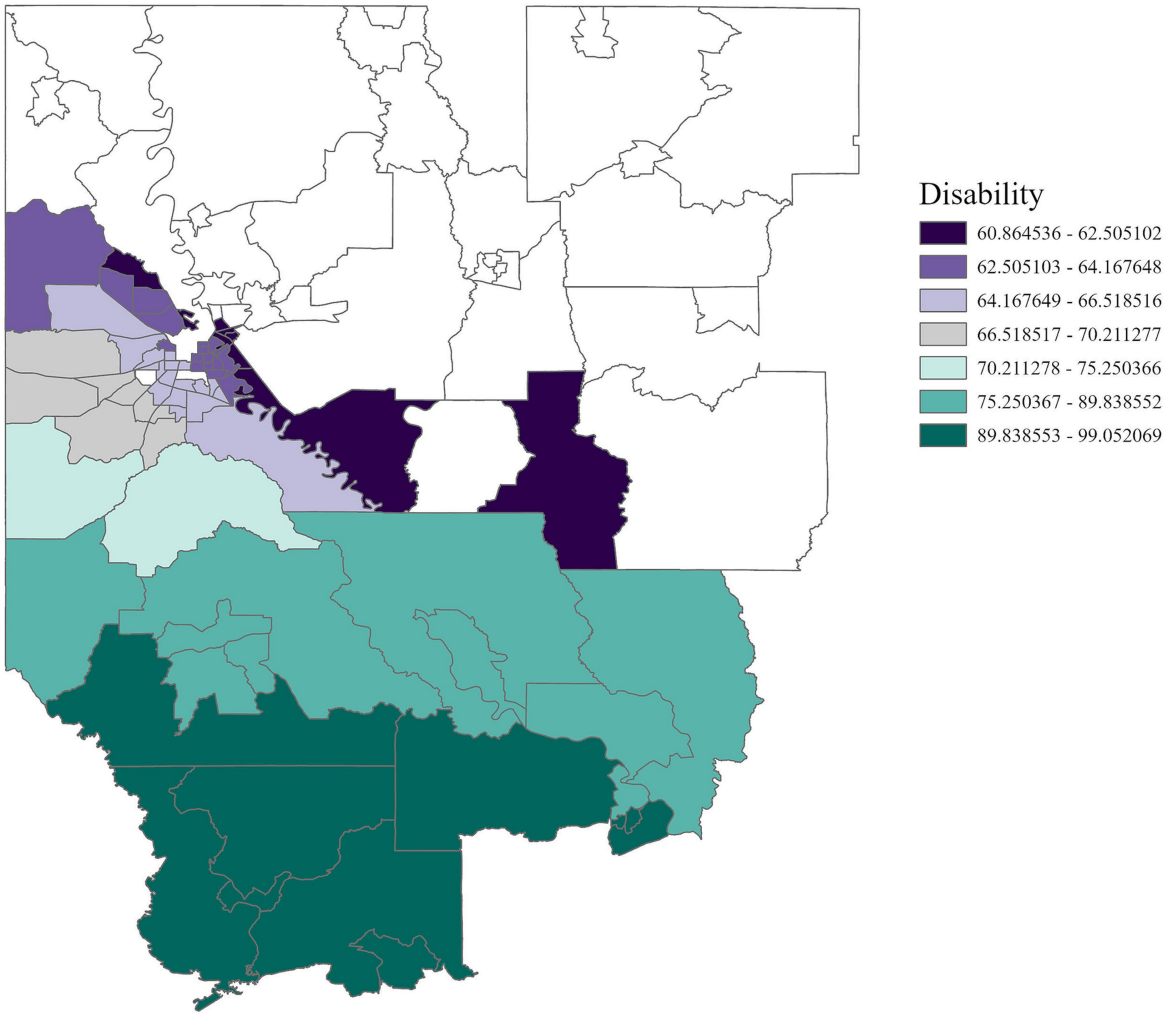
GWR analysis for Region 7, illustrating the spatial variation in the regression coefficient for disability status. Disability is significantly associated with cancer incidence, underscoring the need for targeted disability assistance in this region.

**Figure 11:**
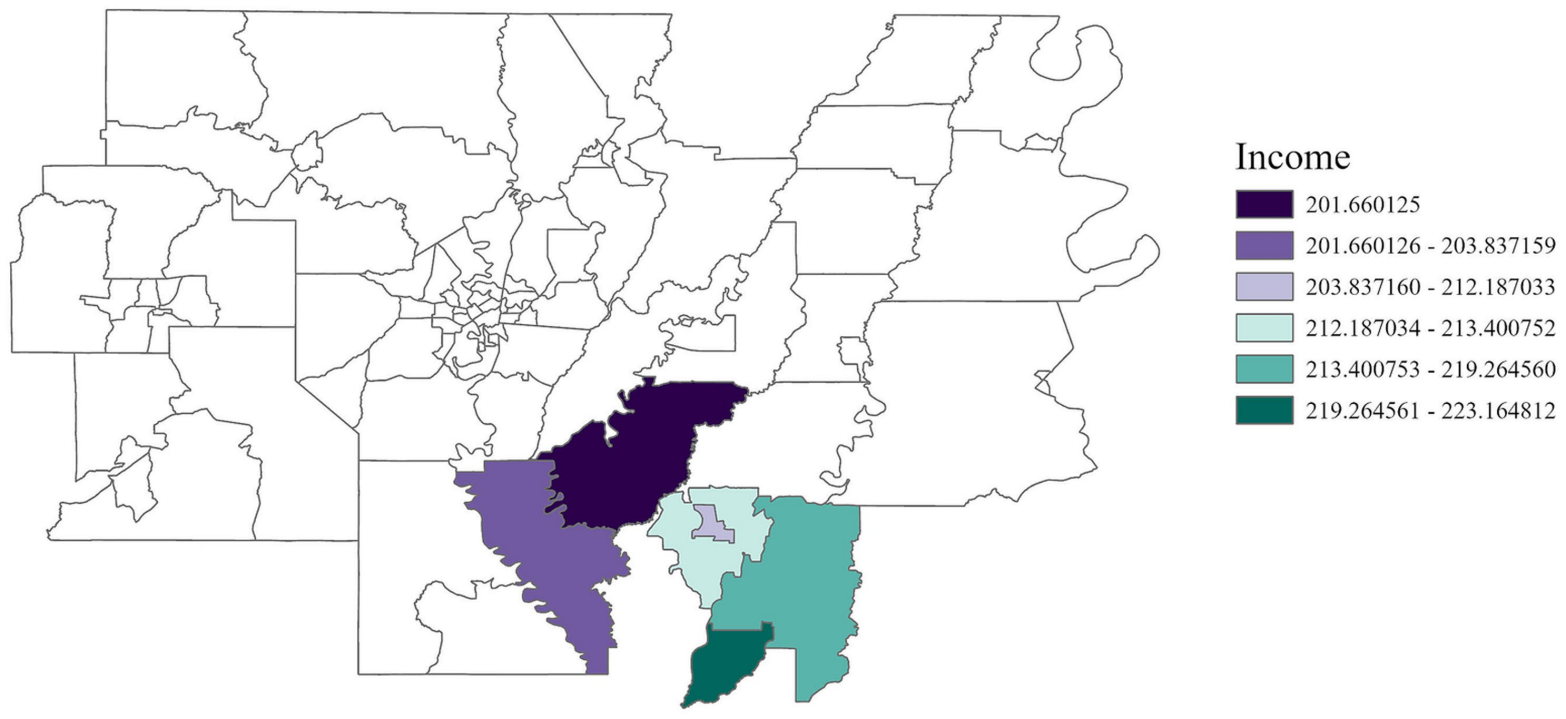
GWR analysis for Region 8, illustrating the spatial variation in the regression coefficient for income. The analysis highlights elevated cancer rates among low-income residents in the southern area of the region.

**Figure 12:**
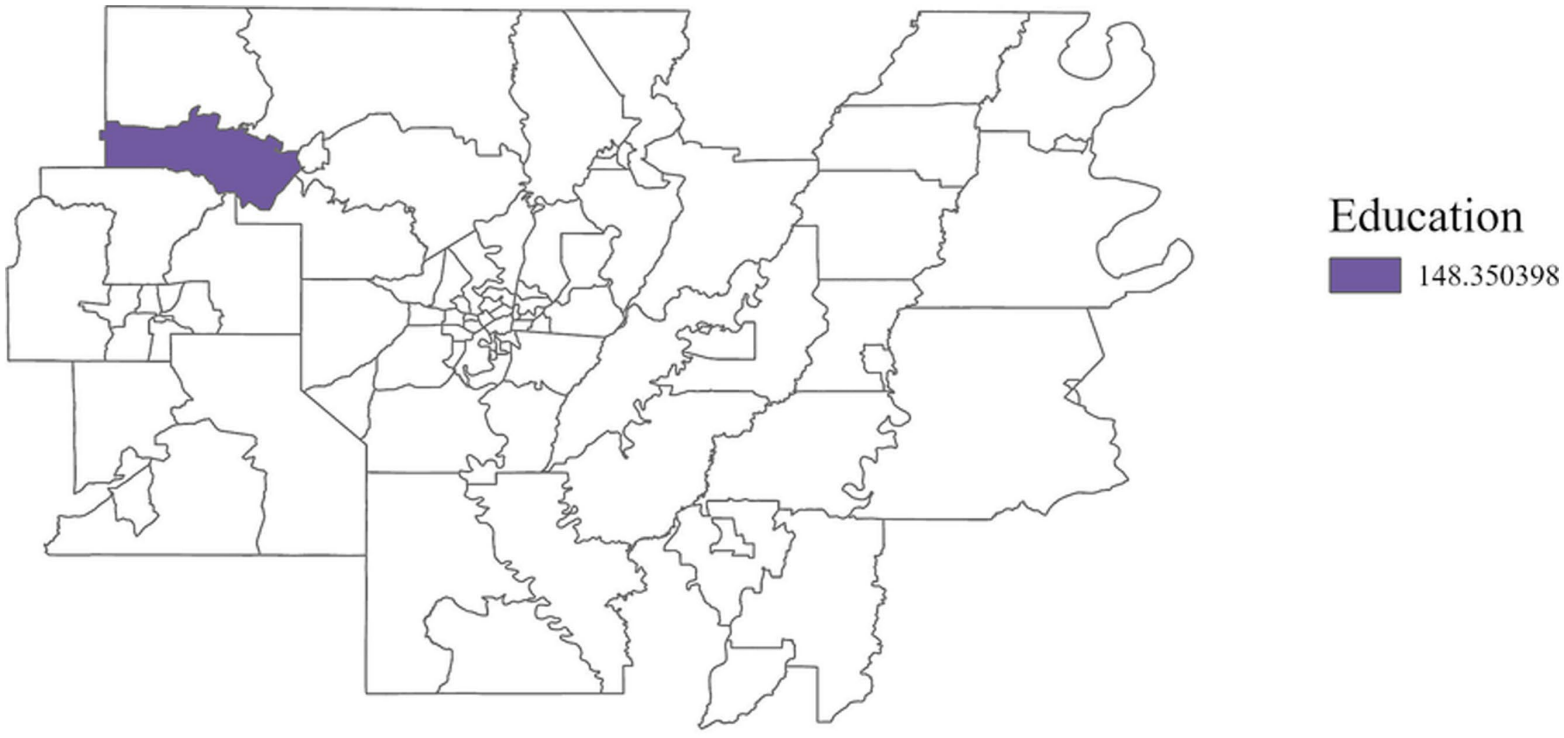
GWR analysis for Region 8, illustrating the spatial variation in the regression coefficient for education. As vulnerability associated with educational attainment increases by one unit, cancer incidence rises by 148.35 cases per 100,000 in this region.

**Figure 13:**
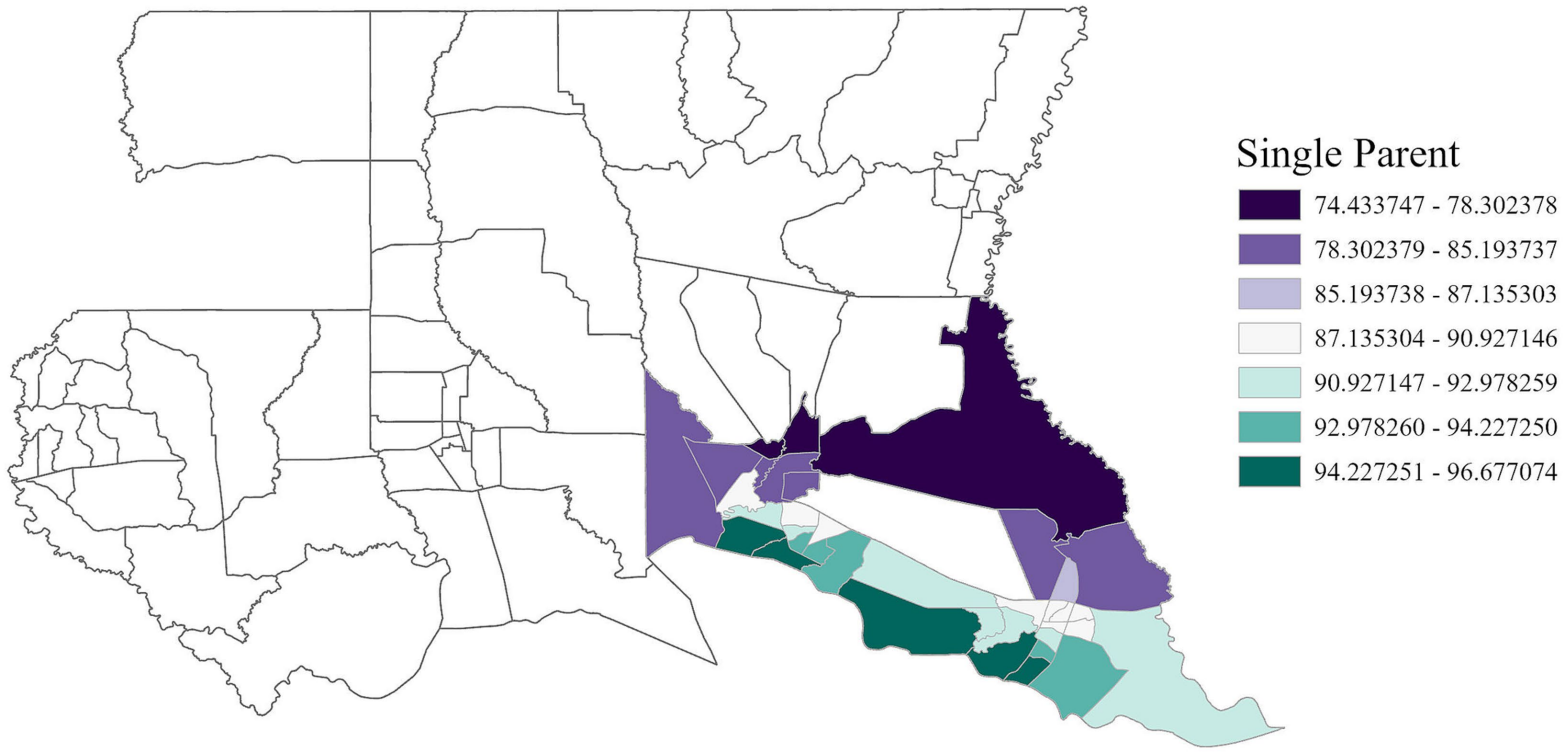
GWR analysis for Region 9, illustrating the spatial variation in the regression coefficient for single parent households. The analysis highlights that household structure is a factor in increased cancer rates, emphasizing the need for targeted support in areas such as financial assistance, childcare, housing and education.

In Region 7, disability status was positively associated with cancer incidence (Figure 10) with an adjusted *R*^2^ of 0.07, emphasizing the heightened risks faced by individuals living with disabilities. Income and education were positively correlated with cancer incidence in Region 8 (Figures 11, 12) (adjusted *R*^2^ = 0.12), which reflects the complex role of economic and education status in shaping cancer outcomes. This association highlights the need for purposeful initiatives aimed at improving both the financial stability and education level of residents in this region. In contrast, race was negatively associated with cancer incidence in Region 8, a relationship that may be influenced by underlying factors not addressed in this study. A significant positive association was observed between single-parent households and cancer incidence in Region 9 (Figure 13), with an adjusted *R*^2^ of 0.15, emphasizing the necessity for targeted interventions within these populations.

### 3.4 Linear regression analysis

The linear regression analysis further explored the relationships between cancer incidence, air toxins, and social vulnerability factors across Louisiana, complementing the GWR results. These analyses confirmed that associations between cancer incidence and environmental or socioeconomic factors vary substantially across different regions, reflecting the interplay between localized exposure patterns and social determinants of health.

#### 3.4.1 Descriptive statistics

Descriptive statistics for all variables included in the linear regression analysis are presented in Table 3. Across Louisiana’s census tracts, the mean cancer incidence rate was 489.56 cases per 100,000 population (SD = 62.28). The average risk of cancer from air toxins was 49.72 cases per million (SD = 34.45), with a minimum of 27.35 and a maximum of 826.31. These figures highlight substantial variability in both cancer incidence and environmental exposure levels across the state.

**Table 3:** Descriptive statistics.

Variables	*N*	Minimum	Maximum	Mean	Std. deviation
Cancer incidence	908	311.60	854.90	489.56	62.28
Air toxins	908	27.35	826.31	49.72	34.45
Poverty	908	0.00	1.00	0.48	0.28
Employment	904	0.01	1.00	0.48	0.28
Income	908	0.00	1.00	0.49	0.28
Education	905	0.00	1.00	0.49	0.28
Disability	908	0.00	1.00	0.49	0.28
Single parent	907	0.00	0.99	0.51	0.28
Minority	908	0.00	1.00	0.47	0.28
Air toxins	908	1.44	2.92	1.67	0.10
Air toxins and poverty	908	0.00	2.46	0.80	0.48
Air toxins and employment	904	0.02	2.02	0.81	0.47
Air toxins and income	908	0.03	2.11	0.81	0.47
Air toxins and education	905	0.12	2.21	0.82	0.46
Air toxins and disability	908	0.00	2.00	0.82	0.47
Air toxins and single parent	907	0.02	2.59	0.85	0.48
Air toxins and minority	908	0.00	2.62	0.80	0.49

Indicators of social vulnerability showed similarly wide ranges. Poverty levels ranged from 0.0018 to 1.00, and measures such as income, education, disability, single-parent households, and minority status also displayed substantial variation, reflecting significant socioeconomic disparities across different communities in Louisiana. These disparities are likely key contributors to variations in cancer incidence observed in the regression analyses.

#### 3.4.2 Regression models

The linear regression analysis revealed significant relationships between cancer incidence and air toxins along with various socioeconomic factors across Louisiana, with regional variation in the results. Table 4 summarizes the significant variables identified in the linear regression analysis and the direction of their associations. Region-specific regression models are detailed in Table 5, including coefficients, standard errors, and confidence intervals.

**Table 4:** Significant variables from the linear regression analysis and the direction of their associations.

Location	Significant variables (+/− Relationship)
Overall Louisiana	Single Parent Households (+)
	Air toxins * Single Parent Households (+)
Region 1	Income (+)
	Air toxins * Education (+)
Region 2	None
Region 3	Education (−)
	Air toxins (−)
	Air toxins * Education (−)
Region 4	Air toxins (−)
	Air toxins * Poverty (−)
	Minority (+)
	Air toxins * Education (+)
Region 5	None
Region 6	None
Region 7	Disability (+)
	Air toxins * Disability (+)
Region 8	None
Region 9	Air toxins (−)
	Single Parent Households (+)

**Table 5:** Regression models for all regions including the regression coefficient.

Variables	*B*	Std. Error	*β*	*t*	*p*	CI
Region 1						
Constant	448.75	9.69		46.32	0.00	(429.63, 467.86)
Income	75.29	18.82	0.28	4.00	0.00	(38.16, 112.43)
Air toxins * Education	49.89	11.57	0.30	4.31	0.00	(27.06, 72.71)
Region 4						
Constant	1252.47	189.96		6.59	0.00	(876.09, 1628.85)
Minority	59.08	21.85	0.27	2.70	0.00	(15.79, 102.37)
Air toxins * Education	41.11	15.44	0.34	2.66	0.00	(10.52, 71.71)
Region 7						
Constant	459.75	11.83		38.87	0.00	(436.28, 483.21)
Disability	58.08	20.81	0.27	2.83	0.00	(17.52, 100.09)
Air toxins * Disability	35.07	12.21	0.28	2.87	0.00	(10.86, 59.29)
Region 9						
Constant	1025.83	181.20		5.66	0.00	(665.61, 1386.05)
Single parent	47.90	23.51	0.22	2.04	0.04	(1.17, 94.63)

Statewide, single parent households along with the interaction between air toxins and single parent households was significant. In Region 1, both income and the interaction between air toxins and education were significant, indicating that higher income areas near industrial sites may still face substantial cancer risks when combined with lower educational attainment. In Region 4, minority status was a significant predictor of higher cancer incidence, and interactions between air toxins and both education and poverty further explained variations in cancer rates. These interactions suggest that cancer incidence is not solely driven by environmental exposure but also shaped by socioeconomic contexts. In Region 7, disability and its interaction with air toxins were positively associated with cancer incidence, highlighting a population subgroup with elevated vulnerability. In Region 9, single-parent households were significantly associated with higher cancer rates, reaffirming the importance of household structure as a determinant of health outcomes. Regions 2, 5, 6, and 8 did not show significant relationships in the linear regression models, which may reflect data limitations or differing regional dynamics.

## 4 Discussion

This study highlights the complex relationship between cancer incidence, environmental exposures, and social vulnerability factors in Louisiana, one of the highest concentrations of industrial sites in the United States. The findings underscore significant regional disparities in cancer incidence, shaped by both airborne toxic exposures and socioeconomic determinants, offering a deeper understanding of how these factors interact to influence health outcomes. The analysis highlights that cancer risk from air toxins is not evenly distributed throughout the state but instead shows significant spatial clustering, particularly in regions like Cancer Alley, where industrial activity is dense. The spatial autocorrelation analysis confirmed that air toxins are highly concentrated in specific areas, suggesting persistent environmental risks for nearby communities. These findings are consistent with prior studies indicating that fence-line communities—neighborhoods adjacent to industrial sites—experience elevated health risks due to sustained exposure to toxic emissions (9, 16).

Louisiana exhibits considerable regional differences in socioeconomic and environmental conditions, which contributes to differences in cancer incidence across the state. Geographic mapping revealed that high cancer rates tend to surround clusters of high cancer risk and power plants. These areas need to be prioritized for policy reform and environmental justice efforts, as residents near these facilities face a greater burden of cancer risk from air pollution.

Both the linear regression and the geographically weighted regression (GWR) analyses highlight the intricate relationship between social vulnerability, air pollution and cancer incidence. At the state level, findings from both analytical approaches consistently identified single-parent households as significantly associated with higher cancer rates, while air toxins, along with their interaction with single-parent households, further amplified cancer incidence. Interestingly, GWR also identified varying relationships with education. In some regions, higher education levels were positively associated with cancer incidence. This could be explained by the fact that more educated communities often have better access to healthcare services, leading to increased detection and diagnosis rates rather than a true increase in cancer occurrence. These results underscore that air pollution and cancer risk are shaped not only by environmental exposure but also by social and economic contexts, a finding echoed in broader research showing that impoverished communities face disproportionate health risks (24, 25). Notably, while air toxins were negatively associated with cancer incidence at the statewide level, this finding likely reflects regional differences in exposure patterns and other unmeasured factors.

Regional differences were evident across the analyses. In Region 1, both analyses showed a significant link between income, education, and cancer rates. Air pollution was positively associated with cancer incidence alone in the GWR as well as in the interaction term with education in the linear regression model, suggesting that this region faces a tremendous burden of air pollution that can be explained by poor educational attainment (23, 26). The GWR revealed a positive spatial correlation between race and cancer rates, emphasizing that this relationship varies across geographic areas. Targeted interventions are needed to address the disproportionate cancer burden experienced by this population and improve health outcomes. The positive association between air toxins and cancer incidence is expected, considering the high concentration of industrial site emissions in this region. Despite being one of the smallest regions in Louisiana, Region 1 experiences some of the highest power plant emissions, underscoring the urgent need for interventions to mitigate the risk of cancer for its residents. The linear regression did not identify any significant variables for Region 2. In the GWR, no significant relationships between the independent and dependent variables were identified for Regions 2 and 3. A negative adjusted *R*^2^ value was found, suggesting that the model may not adequately explain the spatial variability of cancer incidence in these regions. Additionally, the EDA did not specify any significant variables for these regions, which could be due to spatial heterogeneity, emphasizing the need to assess these relationships on a more granular level (e.g., neighborhood or parish level). Other potential explanations include inaccurate or incomplete data from this area surveillance bias, or missing confounding factors like access to healthcare, smoking, and pre-existing health conditions should be included in the analysis.

In Region 3, significant negative associations were found for education, air toxins, and the interaction between air toxins and education in the linear regression analysis. One parish, St. John the Baptist, reported a cancer risk from air toxins of 826.31, ranging from 27.35 to 826.31 in this region. The large range in this variable may have influenced the results. Additionally, the relationship between education, air toxins, and cancer rates is likely complex and may need to be explained by other unmeasured variables, such as healthcare access and lifestyle factors (e.g., physical inactivity, psychosocial stress). These relationships will need to be explored in future studies (NAACP (27)). In Region 4, race was significant in both analyses. The role of minority status, along with the interaction between air pollution and education, highlights the need to address racial disparities and the combined risks of social vulnerability and air toxicity in this region. The connection between minority races and poor health outcomes is well-documented, with these communities being more vulnerable to adverse health effects and less likely to participate in preventative screenings (8, 16, 28). Furthermore, these communities exhibit a disproportionate burden from environmental pollution and often lack adequate access to healthcare (12, 29). The negative association between cancer incidence and the interaction between air toxins and poverty in Region 4 may be explained by higher-income areas being located near industrial zones, where environmental exposures are high. These areas may show unexpected trends due to confounding factors such as urban density, increased health-seeking behaviors, and greater access to healthcare, which may result in more frequent cancer screenings and earlier diagnosis in wealthier communities compared to lower-income areas.

Both analyses showed no significant associations in Regions 5 and 6. This may be due to data limitations, insufficient sample size, or other unmeasured socioeconomic and health factors. These regions had the least amount of data compared to the other regions, which could account for the insignificance. Region 7 showed significant associations between disability in both analyses and the interaction term with air toxins in the linear regression, which shows the combined risks for individuals with disabilities in this area with high air toxin exposure. The GWR further enables the identification of specific areas where individuals with disabilities are most at risk, allowing public health initiatives to target these areas more effectively. Previous research has found a significant relationship between disability status and cancer incidence as well as air pollution (30, 31). Income and education were positively correlated with cancer incidence in Region 8, which is consistent with existing literature that suggests that individuals with lower income and education levels often lack the resources needed to effectively reduce or prevent cancer risk, including access to early cancer screenings, preventative services and knowledge of healthy lifestyle choices (32, 33). Race was negatively associated with cancer incidence in Region 8, warranting further investigation to explore potential driving factors, such as local environmental conditions, genetic factors and social behaviors. Finally, Region 9 exhibited a significant positive relationship between single-parent households and cancer incidence, suggesting that family unit configuration may impact cancer outcomes. The negative association between air toxins and cancer rates for Regions 3, 4 and 9 may be due to the latent effect of cancer. The latency period associated with cancer caused by industrial toxins, specifically lung cancer, may be as long as 20 years. Our study analyzed air toxin data from 2011 and cancer outcomes from 2011 to 2020. Future research should include updated cancer incidence data to examine if the association between air pollution and cancer rates are positively associated in this region.

Overall, both analyses emphasize the significant role of socioeconomic vulnerability and environmental exposure in shaping cancer incidence patterns across Louisiana, with the differences in the strength and direction of these relationships between regions. The analyses demonstrate that economic and social factors are particularly influential in regions with greater social vulnerability. The results suggest that higher cancer rates are linked to greater cancer risk from air toxins among single-parent households, disabled individuals, and people with limited academic achievement. These findings align with previous research that links higher social vulnerability to worse health outcomes, particularly in areas with a high occurrence of environmental stressors (16, 20, 34).

These results underscore the necessity for targeted public health interventions in regions characterized by high environmental pollution and social vulnerability. Policies aimed at implementing stricter emission regulations to reduce dangerous air toxins, particularly in areas like Cancer Alley, are vital. The Clean Air Act requires each state to develop and implement its own State Implementation Plan (SIP) to meet national air quality standards (35). In 2021, the EPA released a report outlining the results of a risk assessment of ethylene oxide-emitting facilities in Louisiana (36). The five facilities identified had a maximum individual lifetime cancer risk up to 700 in one million. Furthermore, Louisiana failed to submit the SIP for the 1-Hour Primary Sulfur Dioxide National Ambient Air Quality Standard in 2013. Stricter policies are needed, focusing on real-time air pollution monitoring to ensure compliance with air quality standards. This would also allow regulators to control excessive emissions, thereby reducing the environmental burden on vulnerable populations. Additionally, regular air pollution education is needed to improve health literacy among fence-line communities and provide updates regarding toxic air emissions to residents. Furthermore, public health initiatives aimed at improving socioeconomic burden in vulnerable regions could help reduce cancer rates in these areas. Programs that enhance the workforce, address housing concerns, provide affordable and quality education, and offer disability support would significantly advance the health and well-being of communities most affected by cancer incidence and air pollution. In addition, disadvantaged areas would benefit from more cancer education and community-level preventative services.

### 4.1 Limitations

There is limited robust and publicly available information and data on other factors influencing cancer incidence, such as other environmental exposures (e.g., water contamination, radiation, occupational hazards, lifestyle factors [e.g., smoking, diet, obesity, alcohol consumption], etc.), and genetic predispositions. Practical research links cancer incidence to smoking and obesity (37), though this data is not available at the census tract level in Louisiana. Future studies should incorporate these additional variables to better understand how these factors and cancer risk from air toxins influence cancer incidence. Furthermore, the differences in data reporting across regions may lead to variations in the outcomes found in this study. Additionally, minute differences may not be captured at the regional level. Future studies should examine parish-level data to identify localized effects. The housing-related SVI theme and limited English proficiency variable, which contained numerous zeros, were excluded from this analysis. As a result, there may be missed explanations for cancer risk due to air toxins in certain areas. Future studies should aim to incorporate these variables to identify the impact of housing conditions and language barriers on cancer outcomes (4, 38, 39).

It is important to clarify that missing data in certain regions may impact the ability to fully characterize the cancer incidence patterns across Louisiana, particularly Region 1. This is due to incomplete or unavailable cancer incidence data for some census tracts. No adjustments were made in the analysis to account for the missing data, which could potentially affect the findings. Future analyses may benefit from obtaining missing data to provide a more comprehensive understanding of the cancer incidence patterns across Louisiana’s regions. Future sensitivity testing or the application of multiple imputation strategies may improve robustness in subsequent analyses.

Additionally, the use of 2011 EPA NATA data introduces a temporal mismatch with cancer incidence data spanning from 2011 to 2020. This may limit the ability to detect effects due to latency in cancer development or changes in industrial emissions over time. Although this dataset was the most comprehensive publicly available source for air toxics at the time, more recent assessments (e.g., 2014 NATA, 2017 Air Toxics Screening Assessment, Toxics Release Inventory [TRI] data, etc.) should be used in future analyses to improve temporal alignment. Longitudinal or time-series studies could also be used to examine trends in exposure and outcomes over multiple decades. Despite these limitations, this study benefits from a large and comprehensive sample size (*n* = 908 census tracts), enabling the detection of significant relationships between air toxins, social vulnerability, and cancer incidence across Louisiana. Nevertheless, future research that integrates updated environmental risk data, behavioral and genetic factors, and more granular geographic units will be essential to refining these findings and informing targeted public health interventions.

## 5 Conclusion

This study provides a comprehensive examination of how the intersection of air toxin exposure and social vulnerability factors shapes cancer incidence in Louisiana. The results demonstrate that cancer risks are concentrated in specific geographic regions, particularly those with high industrial activity, and are amplified by socioeconomic disadvantages, including poverty, low education, minority status, disability, and single-parent households. These findings highlight the multifaceted nature of cancer disparities, where environmental and social determinants interact to produce disproportionate health burdens among marginalized communities.

Combining geospatial and statistical analyses, this study identifies clear patterns of regional variation in cancer incidence and its relationship to environmental exposures and social vulnerabilities. Regions such as Cancer Alley continue to experience heightened cancer risks, especially among communities of color and socioeconomically disadvantaged groups. The results emphasize that environmental pollution alone does not fully explain cancer disparities—rather, the combination of toxic exposures with underlying social and economic vulnerabilities significantly exacerbates health risks.

The evidence presented underscores an urgent need for targeted, multidimensional public health and policy responses. First, stronger environmental regulations are needed to reduce hazardous air emissions, particularly in areas with high cancer risks. Real-time air quality monitoring and enforcement mechanisms must be established to ensure industrial compliance and provide residents with timely information. Second, addressing the social determinants of health is crucial in mitigating the broader factors that contribute to cancer risk. Interventions must focus on improving access to preventive healthcare, education, and economic opportunities, especially in regions with high social vulnerability. Programs designed to increase cancer screening, early detection, and education on environmental health risks are critical in reducing cancer incidence among the most affected populations. Third, community engagement is crucial to ensure that interventions are culturally relevant and tailored to meet community needs. Partnerships with local organizations, healthcare providers, and advocacy groups can facilitate more effective outreach and build trust among historically marginalized residents in environmental and health policy decisions. Ultimately, this study recommends the development of enhanced data collection and monitoring systems to ensure that public health responses are grounded in accurate and up-to-date information. Improved surveillance of cancer incidence and environmental exposures at more granular geographic levels, including census tracts and neighborhoods, would enable the more precise identification of at-risk communities and the development of tailored interventions.

In conclusion, reducing cancer disparities in Louisiana requires an integrated strategy that simultaneously addresses environmental exposures and social inequities. In addition to regulatory and health interventions, targeted data improvements, including more granular geospatial coverage and updated exposure assessments, are essential to strengthen surveillance and equity-focused response strategies. By adopting a holistic approach that combines regulatory action, social support, and community-based solutions, policymakers and public health officials can make meaningful progress in protecting vulnerable populations and advancing health equity across the state.

## Data Availability

The original contributions presented in the study are included in the article/supplementary material, further inquiries can be directed to the corresponding author.
